# Medical Schools in Russia

**Published:** 1849-03

**Authors:** 


*Medical Schools in Russia.*—The medical schools of Russia are carried on in a grand and becoming manner, they are plentifully supplied in every respect. The studies must extend over five years, each year beginning in August, and terminating in June. The whole of May is given up to examinations; all the courses last the full year, and every student is obliged to attend them, irrespective of the peculiar branch of medical science he may wish to study. There are six *censures*, or examinations. If the candidate do not give satisfaction with the first three, he is put back for one year; but when he has creditably passed five, he obtains his doctor's degree. The sixth is only attempted by those who are looking for state appointments. The fourth and fifth are especially dedicated to clinical instruction; after which the young men may settle in any part of the empire.—*London Lancet.*

